# Vibration Perception Threshold as a Method for Detecting Diabetic Peripheral Neuropathy: A Systematic Review of Measurement Characteristics

**DOI:** 10.3390/diagnostics16020217

**Published:** 2026-01-09

**Authors:** Danijela Ribič, Nejc Šarabon

**Affiliations:** 1 Faculty of Health Sciences, University of Primorska, Polje 42, SI-6310 Izola, Slovenia; 2 Ludwig Boltzmann Institute for Rehabilitation Research, A-1090 Vienna, Austria

**Keywords:** vibration perception threshold, diabetic peripheral neuropathy, screening test, quantitative sensory testing, diagnostic validity

## Abstract

**Background:** Diabetic peripheral neuropathy (DPN) is one of the most common complications of diabetes mellitus (DM), leading to sensory loss, balance disturbances, and an increased risk of ulcers and amputations. Early screening is crucial, and devices for measuring vibration perception threshold (VPT) play an important role in the timely detection and management of this condition. **Objective:** The aim of this systematic review was to evaluate the diagnostic accuracy and reliability of VPT measurement devices in individuals with DM. **Methods:** A systematic search was conducted in four databases, including studies that assessed the diagnostic accuracy and reliability of VPT measurement devices in patients with type 1 or type 2 DM, with VPT compared against reference standards for DPN, including nerve conduction studies (NCS) and clinical diagnosis. Cross-sectional and case–control studies were included. Risk of bias was assessed using the Quality Appraisal of Reliability (QAREL) tool and the JBI Critical Appraisal Checklist for Diagnostic Test Accuracy Studies. **Results:** Eighteen studies were analyzed. Most studies demonstrated moderate sensitivity and specificity and an acceptable level of reliability, with results varying according to technical and methodological factors. **Conclusions:** VPT measurement devices appear to be useful screening tools for detecting DPN; however, their diagnostic accuracy and reliability are not uniform and largely depend on technical and methodological factors. Standardized threshold values and measurement procedures, along with further research comparing the effectiveness of different protocols, are needed to improve clinical utility.

## 1. Introduction

The global burden of diabetes mellitus (DM) is rising rapidly, and epidemiological forecasts suggest that the number of people living with the disease may approach approximately 438 million by 2030 [1]. With the rising prevalence of DM, the incidence of its chronic complications is also increasing, among which diabetic peripheral neuropathy (DPN) is particularly concerning. DPN leads to sensory loss in the extremities, which may result in balance and gait disturbances as well as the development of pressure ulcers and subsequent infections [2]. DPN represents one of the leading causes of diabetes-related morbidity and is a major contributor to reduced quality of life, increased healthcare utilization, and preventable lower-limb amputations.

DPN results from pathological changes in peripheral nerve structure and function that develop over time in the context of chronic hyperglycaemia and metabolic imbalance [2]. The condition most commonly affects distal regions, including the lower extremities and hands, in a pattern referred to as the “stocking–glove” distribution [3]. It is estimated that DPN is present in approximately 50% of individuals with DM of more than 10 years’ duration and in 10–30% of patients already at the time of DM diagnosis [4]. Disease progression is further influenced by inadequate glycemic regulation, abnormal lipid profiles, and elevated blood pressure, all of which complicate management and increase the likelihood of serious complications [2]. Owing to the high prevalence of DPN and its serious functional consequences, early detection of DPN and the implementation of appropriate preventive measures are essential components of DM management [5].

Insufficient or delayed detection of DPN significantly contributes to the development of foot complications, which are common among patients with DM. Reported rates indicate that diabetic foot ulcers develop in approximately 1.0–4.1% of individuals each year, while cumulative lifetime risk has been estimated to approach 25% [6]. Given these data, early screening and timely identification of DPN are of paramount importance, as prompt intervention can substantially reduce the incidence of ulcers and amputations [7]. These severe complications highlight the need for accurate and timely diagnostic approaches for DPN. A range of diagnostic methods is available for identifying DPN, including nerve conduction studies (NCS), quantitative sensory assessments, and structured clinical evaluations. Although NCS are widely regarded as the reference standard because of their objective and reproducible results, their high cost and dependence on specialized equipment restrict routine implementation, particularly in settings with limited resources [8].

To address these limitations, simpler screening methods have also been developed and are recommended by the International Diabetes Federation, the International Working Group on the Diabetic Foot, and the American Diabetes Association. These organizations propose several bedside (chairside) tests, including the 10 g monofilament test, a 128 Hz tuning fork, the Ipswich Touch Test, temperature sensation testing, vibration perception threshold (VPT), pain sensation testing, proprioception testing, and ankle reflex assessment [9,10,11]. These tests are easy to perform, inexpensive, and suitable for bedside application [6].

In the assessment of vibration sensation, the standard 128 Hz tuning fork has traditionally been used as a simple method for determining the presence of vibratory perception [12]. However, this instrument does not provide quantitative information on the degree of vibration perception loss. Quantitative vibration testing is important, as reduced vibration perception has been identified as a predictive factor for the risk of foot ulceration [13]. Consequently, a number of electromechanical instruments have been introduced to enable quantitative evaluation of vibration perception. These devices offer advantages such as continuous delivery of vibratory stimuli and a wider range of vibration intensities [14].

VPT measurement is performed using a device that gradually increases electrical voltage (0–50 V) to generate vibrations. The patient is seated in a relaxed position, typically with eyes closed to prevent visual perception of the stimulus. The probe is gently applied to the skin, most commonly to the pulp of the great toe, but it may also be placed on the metatarsal heads or the plantar arch. The stimulus is gradually increased until the patient perceives the vibration and verbally confirms its detection (e.g., by saying “yes”). The procedure is repeated three times, and the result is the average of the three measurements. Additionally, a sham (no-vibration) test may be included to verify the participant’s ability to reliably distinguish the presence of the stimulus [15].

One of the major challenges in the clinical application of VPT is the lack of consensus regarding diagnostic cut-off values for DPN. Despite the widespread use of VPT measurement, optimal cut-off values for the diagnosis of DPN have not been consistently established. Some studies use a VPT of ≥25 V [16,17] as one of the diagnostic criteria for DPN in patients with DM, whereas others apply a lower threshold of ≥15 V [18]. These VPT cut-off values have been defined based on different reference standards used to diagnose DPN, such as NCS, the Neuropathy Disability Score (NDS), or the Michigan Neuropathy Screening Instrument (MNSI).

As VPT measurement devices are used as screening tools for the early detection and monitoring of DPN, knowledge of their diagnostic accuracy and reliability is essential for correct clinical interpretation of results and for the selection of appropriate screening instruments in clinical practice. Nevertheless, the evidence base regarding the diagnostic performance and reliability of these devices remains limited. As a result, clinicians and researchers lack clear guidance on the diagnostic accuracy and reliability of VPT devices used in different clinical contexts. To date, only one literature review addressing VPT measurement has been published [19]; however, this review does not apply clearly defined reference standards for DPN diagnosis, does not systematically evaluate diagnostic accuracy and reliability, and provides limited critical discussion of the heterogeneity among included studies. Moreover, it does not incorporate more recent research. Therefore, the aim of this systematic review is to evaluate the diagnostic accuracy and reliability of VPT measurement devices used for the detection of DPN.

## 2. Materials and Methods

### 2.1. Information Sources and Search Strategy

We conducted a systematic review of the existing literature on VPT as a method for detecting DPN, focusing on the key measurement properties of the method, namely diagnostic accuracy and reliability. The PRISMA 2020 checklist was used as a reporting guide for this systematic review (see Supplementary Materials). Articles were searched in the PubMed, Scopus, CINAHL, and MEDLINE databases. The last search was performed on 3 November 2025. The following English keywords were used: (vibration OR “vibration perception threshold” OR neurothesiometer OR biothesiometer) AND (neuropathy) AND (diabetic) AND (reliability OR reproducibility OR repeatability OR accuracy OR sensitivity OR specificity OR “diagnostic validity”).

The keywords were required to appear in the title, abstract, or keyword list of each article. Reference lists of the included studies were also examined manually to identify relevant pertinent publications. After duplicate removal, the studies were screened by D.R., and the eligibility of the included studies was subsequently verified by N.Š. This systematic review was prospectively registered in the PROSPERO database (CRD420251186301).

Studies were included if they met the PICOS eligibility criteria. Articles not published in English and those not available in full text were excluded. Studies with a retest interval longer than one year were also excluded, as disease progression could have influenced the results. Studies evaluating the measurement properties of 128 Hz tuning forks were not included, as these instruments are used for qualitative assessment of the presence or absence of vibration sensation and do not allow quantitative determination of VPT. The relevance of records was initially assessed based on titles, followed by abstract screening. A qualitative synthesis of the included studies was performed.

### 2.2. Study Eligibility Criteria

Study eligibility criteria were defined according to the PICOS framework [20] as follows:

- P (Population): Women and men of different age groups with type 1 or type 2 DM.
- I (Index test): VPT, expressed in volts (V).
- C (Comparator): Reference standards for DPN (e.g., clinical diagnosis, NCS).
- O (Outcomes): Diagnostic accuracy (sensitivity, specificity, AUC) and reliability (inter-rater reliability and intra-rater reliability).
- S (Study design): Case–control and cross-sectional studies.

### 2.3. Study Quality Assessment

The methodological quality of the included studies was evaluated by D.R. using the Quality Appraisal of Reliability (QAREL) tool for reliability studies and the Joanna Briggs Institute (JBI) Critical Appraisal Checklist for Diagnostic Test Accuracy Studies. The quality assessments were independently verified by N.Š.

### 2.4. Data Extraction and Analysis

Relevant studies were screened, and those meeting the predefined inclusion and exclusion criteria were included in the review. Data extraction focused on key methodological characteristics and outcome measures to ensure a structured synthesis of the findings. Extracted data included the author(s) and year of publication, study design, and participant characteristics, such as sample size, sex, age, type of DM, duration of DM, and the proportion of participants with DPN. Additional extracted data included the measurement instrument used, measurement site, reference standard, and results related to diagnostic accuracy and reliability. Data extraction was performed by D.R. and independently checked by N.Š.

Sensitivity and specificity values were interpreted according to the following thresholds: values ≥ 90% were classified as high, values between 70% and 89% as moderate, and values < 70% as low [21]. Interpretation of the area under the receiver operating characteristic curve (AUC) followed the criteria outlined by Çorbacıoğlu and Aksel (2023) [22]. An AUC of 0.5 indicates no diagnostic discrimination. AUC values ranging from 0.5 to 0.7 reflect low diagnostic accuracy, whereas values between 0.7 and 0.8 are considered indicative of moderate performance. AUC values in the range of 0.8–0.9 are interpreted as demonstrating good diagnostic accuracy, while values exceeding 0.9 up to 1.0 are indicative of excellent performance. An AUC of 1.0 represents perfect diagnostic accuracy [22]. Cohen’s kappa coefficient was interpreted according to the classification proposed by McHugh (2012) [23]: values between 0.00 and 0.20 indicate no reliability; 0.21–0.39 minimal reliability; 0.40–0.59 weak reliability; 0.60–0.79 moderate reliability; 0.80–0.90 strong reliability; and values above 0.90 indicate almost perfect reliability. In line with the conservative thresholds recommended by McHugh for health research and clinical practice, kappa values above 0.60 were considered acceptable [23].

The coefficient of variation (CV), was interpreted so that lower values indicate higher reliability. The intraclass correlation coefficient (ICC) was interpreted according to the guidelines of Portney and Watkins (2009), where values above 0.75 indicate good reliability, values between 0.50 and 0.75 indicate moderate reliability, and values below 0.50 indicate poor reliability [24]. Spearman’s correlation coefficient was interpreted based on the classification proposed by Prion and Haerling (2014): values between 0.00 and 0.20 indicate negligible association; 0.21–0.40 weak association; 0.41–0.60 moderate association; 0.61–0.80 strong association; and 0.81–1.00 very strong association [25].

## 3. Results

**Figure 1:**
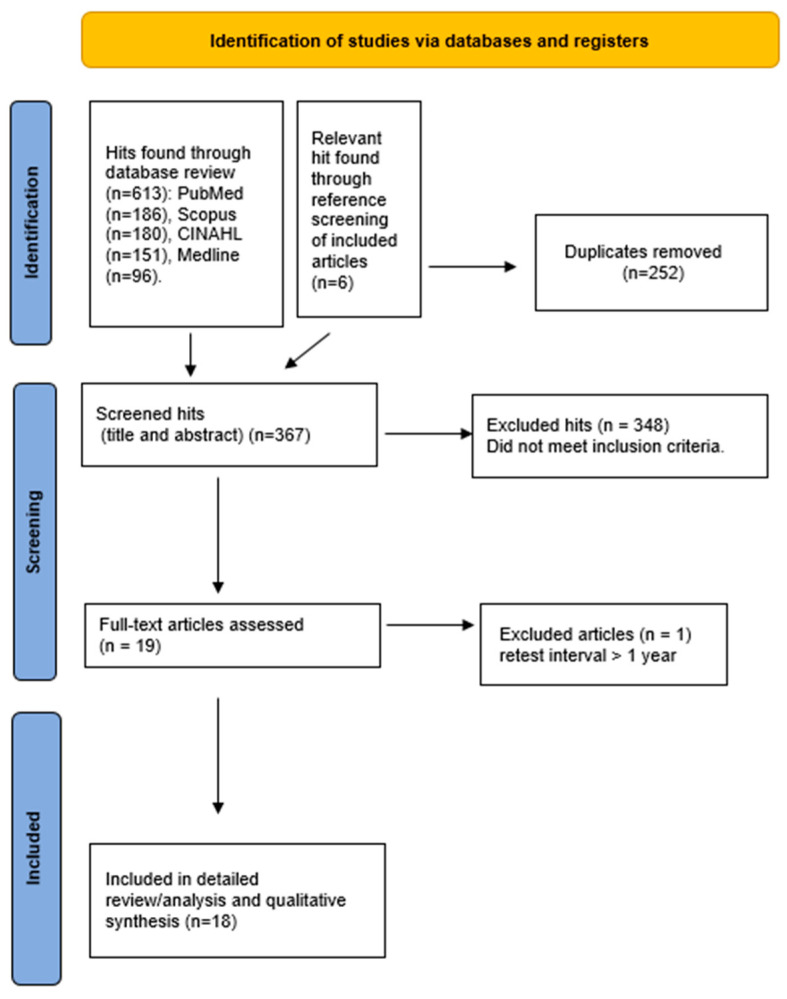
Process of obtaining research with the PRISMA diagram.

The study selection process is shown in the PRISMA flow diagram (Figure 1). After duplicates were removed, 367 records were identified and screened by title and abstract. Following the initial screening, 252 records were excluded for not meeting the inclusion criteria. Full-text evaluation of the remaining publications resulted in the inclusion of 18 studies, published between 1985 and 2024, in the qualitative synthesis.

### 3.1. Risk of Bias in Studies Assessing Diagnostic Accuracy

The risk of bias in the included studies was assessed using the JBI Critical Appraisal Checklist for Diagnostic Test Accuracy Studies. Only one study [26] reported consecutive patient inclusion, while the sampling method was unclear in most of the remaining studies (Table 1). Four studies employed a case–control design [6,27,28,29], which is associated with an increased risk of spectrum bias. All studies applied predefined diagnostic thresholds and used nerve conduction studies (NCS) as the reference standard. Blinding was rarely explicitly reported, and the interval between the index test and the reference standard was generally appropriate.

### 3.2. Risk of Bias in Studies Assessing Reliability

The risk of bias in studies assessing reliability was evaluated using the Quality Appraisal of Reliability (QAREL) checklist. All included studies involved appropriate participant samples, applied the index test correctly, and used suitable statistical measures of agreement (Table 2). However, the risk of bias varied across studies. In one study, raters were not blinded to previous measurement outcomes [31], while in another study, raters were not blinded to clinical information that was not part of the testing procedure [32]. In four studies, raters were not blinded to additional information unrelated to the index test [33,34,35,36]. Consequently, the findings of these studies should be interpreted with consideration of these potential sources of bias.

### 3.3. Characteristics of Included Studies

Eighteen studies were included in this systematic review, collectively reporting on 2346 participants (Table 3). The mean age of participants ranged from 13 to 73 years, with an overall age range of 8–83 years [31,35]. In three studies, age was not reported [33,38,40]. Sex was reported in 14 studies; overall, there were more male participants (*n* = 1206, 51%) than female participants, while sex was not reported in four studies [30,33,38,40]. Participants with type 1 DM [28,31], type 2 DM [5,15,26,27,29,30,34,35], or both types [36,37,39,40] were included. Three studies did not report the type of DM [32,33,38]. The duration of DM was reported in years, with mean values ranging from five to 22 years and an overall range of 0–42 years. In six studies, DM duration was not reported [6,29,32,33,35,40]. DPN was confirmed in 51% of participants (range: 3–100%) in 14 studies, while prevalence data were not reported in four studies [29,34,36,40].

Eight studies reported the sensitivity and specificity of VPT measurement devices [5,6,15,26,27,28,29,30]. Two studies assessed inter-rater reliability [32,40], six studies assessed intra-rater reliability [31,33,34,36,38,39], and two studies assessed both types of reliability [35,37] (Table 4, Table 5 and Table 6).

Diagnostic accuracy was reported as sensitivity and specificity percentages and AUC values. Reliability was assessed using Cohen’s kappa statistics [31,35,37,40], coefficient of variation (CV) [33,36,38], Spearman’s correlation coefficient [39], and intraclass correlation coefficient (ICC) [32,34].

### 3.4. Diagnostic Accuracy of VPT

Eight studies evaluating the diagnostic accuracy of VPT measurement devices were included in the systematic review (Table 4). The devices used were the Biothesiometer [6,26,27,28,30], Vibrasense [5], Neurothesiometer [15], and Sensiometer [29]. Six studies compared VPT values with NCS [5,26,28,29,30]. One study compared VPT with a clinical diagnosis of DPN based on the following criteria: Michigan Neuropathy Screening Instrument (MNSI) symptom score ≥ 4, or MNSI sign score ≥ 2 and Neuropathy Disability Score (NDS) ≥ 6, or NDS ≥ 3 and MNSI symptom score ≥ 4 [27]. Liu et al. (2021) compared VPT values with three reference standards: (1) physician-diagnosed DPN, (2) NCS, and (3) confirmed DPN defined as the presence of neuropathic symptoms combined with abnormal NCS [15].

Five studies reported the diagnostic accuracy of the Biothesiometer. Davis et al. (1997) defined the cut-off value as the 97th percentile of the vibration threshold in a healthy control population and reported moderate sensitivity (80%) and specificity (76%) [28]. Subramani et al. (2024) defined a cut-off value of VPT > 25 V for the presence of neuropathy and reported moderate sensitivity (70%) and specificity (87%) [6]. Hu et al. (2021) defined a cut-off value of VPT > 14.05 V and reported moderate sensitivity (80%) and high specificity (92%) [27]. The area under the ROC curve (AUC) was 0.941, indicating excellent diagnostic performance [27]. Ramanathan et al. (2021) evaluated diagnostic accuracy at VPT > 15 V and VPT > 25 V. At VPT > 15 V, they reported moderate sensitivity (78.6%) and low specificity (52.9%), whereas at VPT > 25 V, they reported low sensitivity (50%) and high specificity (91.2%) [30]. Mythili et al. (2010) used a cut-off value of VPT > 15 V and reported moderate sensitivity (86%) and specificity (76%) [26].

Sharma and Kumar (2023) reported moderate sensitivity (82%) and specificity (79%) for the Vibrasense device using a cut-off value of VPT ≥ 15 V. The AUC analysis (AUC = 0.84) indicated good diagnostic discrimination [5]. Liu et al. (2021) used the Neurothesiometer and defined a cut-off value of VPT > 14.9 V [15]. They reported low sensitivity (67%) and moderate specificity (77–85%). The AUC values (0.76–0.80) indicated fair diagnostic performance [15]. Bharathi et al. (2018) reported moderate sensitivity (70%) and specificity (87%) for the Sensiometer using a cut-off value of VPT > 25 V [29].

### 3.5. Reliability of VPT

Inter-rater reliability was assessed in four studies using different devices, including the Biothesiometer [32,37], Neurothesiometer [35,40], and Maxivibrometer [32] (Table 5). One study reported good inter-rater reliability for the Biothesiometer (ICC = 0.927) [32], while another reported moderate reliability (κ = 0.58–0.65) [37]. Two studies [35,40] reported weak to moderate inter-rater reliability (κ = 0.51–0.61) for the Neurothesiometer. Van Deursen et al. (2001) also reported good inter-rater reliability for the Maxivibrometer (ICC = 0.96) [32].

Intra-rater reliability was assessed in eight studies using various devices, including the Biothesiometer [31,36,37,38,39], Neurothesiometer [33,35,36], and Vibratron II [33,34] (Table 6). Two studies reported weak to moderate intra-rater reliability for the Biothesiometer (κ = 0.51–0.70) [31,37], two reported high reliability (CV = 8.6–18.6%) [36,38], and one demonstrated a very strong correlation (r = 0.91) [39]. Intra-rater reliability of the Neurothesiometer was evaluated in three studies, with one reporting weak to moderate reliability (κ = 0.51) [35] and the remaining two showing excellent reliability (CV = 6–8.1%) [33,36]. Intra-rater reliability findings for the Vibratron II ranged from moderate (CV = 31%) [33] to excellent across the included studies [34].

## 4. Discussion

The aim of this systematic review was to evaluate the diagnostic accuracy and reliability of devices used to measure VPT for the detection of DPN. Most studies demonstrated moderate sensitivity and specificity of VPT measurement devices, along with an acceptable level of reliability; however, the results varied depending on technical and methodological factors.

### 4.1. Diagnostic Accuracy of VPT Measurement Devices

The analysis of eight studies assessing the diagnostic accuracy of VPT measurement devices showed relatively consistent findings. Most studies reported moderate sensitivity and specificity for the diagnostic devices. Sharma and Kumar (2023) reported that the sensitivity (82.14%) and specificity (78.79%) of the Vibrasense device, compared with abnormal NCS findings, were clinically acceptable, particularly considering that VPT measurement is used as a screening tool [5]. The authors further noted that the cost of DPN screening using Vibrasense in their clinical setting was approximately one-tenth of the cost of NCS. Such low testing costs, combined with acceptable diagnostic accuracy, enable healthcare professionals to screen a larger number of patients more efficiently and cost-effectively. Nevertheless, notable differences between study results were observed and warrant further explanation.

One potential source of variability relates to the technical characteristics of the devices used. In the included studies, various devices were used to measure the vibration perception threshold (VPT); however, they were comparable in terms of their basic measurement principle. Across all devices, the vibratory stimulus was generated within a voltage range of 0–50 V, with the voltage gradually increased from the lowest value, and the transition from the absence of vibration to the perception of vibration defined as the vibration perception threshold. However, important methodological differences were observed between studies. Only two studies reported the rate of voltage increase in detail, with Davis et al. (1997) and Ramanathan et al. (2021) specifying a gradual increase at a constant rate of 1 V/s, whereas this parameter was not clearly defined in the remaining studies [28,30]. The devices also differed in vibration frequency, with some operating at 100 Hz [26,28,29] (and others at a higher frequency of 120 Hz [5,30], which may influence vibration perception and contribute to variability in VPT measurements. An additional limitation of most devices is their reliance on manual control by the examiner, particularly with respect to probe application and pressure control, which may affect the repeatability and reliability of the measurements.

Heterogeneity in the reference standards used to compare VPT results was evident across studies. Most studies used NCS as the reference standard, while others relied on clinical scales or combinations of clinical signs and symptoms [15,27]. Liu et al. (2021) demonstrated that the diagnostic performance of VPT was highest when clinically diagnosed DPN—defined as the presence of neuropathic symptoms together with at least one abnormal clinical sign—was used as the reference standard [15]. In contrast, the lowest AUC value (0.761) was observed when NCS served as the gold standard [15]. Conversely, Martin et al. (2010) reported the highest diagnostic performance of VPT when confirmed DPN was used as the reference standard (AUC = 0.800), while the lowest AUC was observed for clinically diagnosed DPN (AUC = 0.745) [41]. These discrepancies may be attributed to differences in DM type among participants and to the use of different VPT devices [41,42].

Despite inconsistent findings, commonly recommended and widely used VPT values are based on NCS as the gold standard [3,35,43]. However, many patients initially present with small-fiber neuropathy symptoms and signs without abnormal NCS findings [44]. Abnormal NCS typically reflects a later stage of DPN [45]; therefore, using NCS as the gold standard may result in higher VPT cut-off values, potentially causing early cases of DPN to be missed and reducing diagnostic effectiveness.

Differences in diagnostic accuracy can also be attributed to variability in selected VPT cut-off values. Across studies, pathological VPT ranged from >14.05 V to >25 V, which substantially influenced sensitivity and specificity. Higher VPTs were associated with improved specificity but reduced sensitivity [5,15,30,46,47]. For example, Sharma and Kumar (2023) demonstrated lower sensitivity (57.14%) at a diagnostic threshold of 25 V compared with 82.14% at 15 V [5]. Similarly, Liu et al. (2021) identified an optimal VPT of >14.9 V, yielding a sensitivity of 66.5% and specificity of 77% compared with NCS, whereas a threshold of 25 V resulted in lower sensitivity (48.4%) but higher specificity (92.5%) [15]. Thus, higher thresholds may lead to under detection of early DPN cases with mild vibration perception impairment.

The VPT of 25 V has been used in some studies due to its ability to predict the development of foot ulcers [36,43,48]. Moreover, age has been shown to be an important modifying factor when defining pathological VPTs. Physiological VPT values increase with age, potentially requiring higher diagnostic thresholds in older populations [15,49]. Liu et al. (2021) reported higher optimal diagnostic thresholds in individuals aged ≥65 years (23.0 V or 25.9 V) compared with those aged <65 years (12.7 V or 12.8 V), regardless of the reference standard used [15]. Additionally, previous studies have demonstrated a negative association between DPN severity and VPT sensitivity [50,51].

One of the most commonly used screening tools for DPN is the 10 g monofilament test. Compared with VPT, this test shows lower sensitivity but higher specificity for DPN detection [5,30,52]. As such, the monofilament test is effective in identifying individuals at risk for foot complications [53] and advanced neuropathy [54,55]. However, it may fail to detect early DPN cases that can be identified using VPT testing [56]. Consequently, the International Working Group on the Diabetic Foot (IWGDF) recommends additional vibration perception testing using a tuning fork or biothesiometer when monofilament testing does not reveal loss of protective sensation [12].

Measurements were performed at different sites on the foot across studies. For studies using multi-site protocols, it would be valuable to assess whether single-site testing—typically at the hallux, which is an established protocol—provides comparable diagnostic accuracy [15,27,34,35]. If equivalent accuracy is demonstrated, a single-site protocol could be preferred, as it would substantially reduce testing time for healthcare professionals. Standardization of measurement sites could improve feasibility and comparability across clinical and research settings.

### 4.2. Reliability of VPT Measurements

Overall, VPT measurements obtained using biothesiometers, neurothesiometers, or maxivibrometers demonstrated acceptable reliability. However, four studies involving participants already diagnosed with DPN reported weak to moderate inter- and intra-rater reliability [32,33,37,39], which is concerning, as high consistency would be expected in this population. Detailed examination suggests that differences in reliability stem from both technical and human factors.

Several studies included small sample sizes [33,35,39] limiting statistical power. In one study, assessors were not blinded to previous results, increasing the risk of bias [32]. Additionally, the use of different statistical methods to assess reliability hindered direct comparisons between studies.

Reliability was influenced by device design and testing procedures. A major limitation is that probe pressure is manually controlled, which may introduce inter-examiner variability [31]. Moreover, the time interval between initial testing and retesting may affect results, as very short intervals (<12 h) can lead to short-term learning effects [57]. To ensure reliable measurements, stimulus application must be standardized: the probe should be applied perpendicular to the skin with consistent force, at the same anatomical site, and the skin should not be cold [31]. Importantly, some studies have explicitly quantified probe pressure, reporting that the probe was applied using only its own weight (approximately 3.7 N in biothesiometer-based protocols), to minimise variability and avoid artificially lowering the VPT through excessive pressure [32]. This highlights the importance of controlling probe pressure as a key methodological factor influencing both measurement reliability and comparability across studies.

Van Deursen et al. (2001) also noted variability between devices in the relationship between applied voltage and actual probe displacement, which constitutes the stimulus for sensory receptors [32]. As a result, measurements obtained using different VPT devices may not be fully comparable. Each laboratory should therefore establish its own reference values and consistently use the same device for longitudinal patient monitoring.

Reliability may also be affected by limited examiner training or differences in examiner experience [33,35], as well as inconsistent understanding of test instructions by participants [31,35,37]. Lanting et al. (2020) demonstrated substantially higher intra-rater reliability among experienced examiners (κ = 0.72–0.78) compared with novices (κ = 0.52) [35], highlighting the importance of standardized training in accordance with guidelines [33,58]. Clear and comprehensible instructions for participants are equally important to reduce variability and improve reliability [58].

Participant characteristics also influenced reliability. Louraki et al. (2014) reported that children and adolescents with type 1 DM, although mostly asymptomatic, exhibited higher VPT values at all measurement sites compared with controls, with statistically significant differences [31]. These findings suggest that early nerve damage can already be detected in childhood and adolescence. However, lower kappa values in this group indicated greater measurement variability, suggesting that VPT assessment may be more reliable in individuals without nerve damage. Similarly, lower reliability was observed in subgroups with poor metabolic control or longer disease duration, potentially due to impaired vibration perception associated with hypoesthesia.

Height and age are important factors influencing vibration perception. Taller individuals have longer peripheral nerves, which may prolong signal conduction and reduce vibration perception [31,59]. Age is a particularly important determinant of VPT [60,61], as normal aging is associated with a decline in peripheral nerve function [62,63]. Higher body mass index may further reduce vibration sensitivity by attenuating stimulus transmission through adipose tissue [64]. As age independently affects neurological and cognitive function, age-specific differences in test reliability may also occur [58].

Although this systematic review demonstrated that VPT measurement devices exhibit acceptable diagnostic accuracy and reliability, they are not yet widely implemented in primary care. Barriers to routine clinical use include the large size of some devices, the need for dedicated space and continuous power supply, and the time required to perform the test [50]. In contrast, devices such as Vibrasense address many of these limitations, as they are portable, lightweight, and battery-powered [5].

### 4.3. Clinical Implications

The findings of this systematic review indicate that VPT measurement devices provide clinically acceptable diagnostic accuracy and reliability for detecting DPN, particularly as a low-cost and accessible screening tool in primary and secondary care. Compared with NCS, VPT enables screening of larger patient populations at lower cost. Importantly, VPT complements the 10 g monofilament test by detecting early DPN cases that may otherwise be missed, in line with international guideline recommendations. This review highlights the need for age- and population-specific diagnostic thresholds, standardized testing procedures, and adequate examiner training to improve reproducibility in routine practice. Portable, battery-powered devices such as Vibrasense further enhance the feasibility of VPT use across diverse clinical settings. Overall, the findings support the integration of VPT measurement devices into routine diagnostic pathways for early detection and monitoring of DPN, facilitating timely preventive and therapeutic interventions and reducing the risk of foot complications.

### 4.4. Limitations

This systematic review has several limitations. The included studies were methodologically heterogeneous, employing different reference standards and VPTs, which limits comparability. Some studies had small sample sizes, used different statistical approaches, and did not consistently blind assessors, increasing the risk of bias. Test reliability was influenced by device characteristics, manual pressure application, examiner experience, and participant-related factors such as age, height, and body mass index. Studies included participants with both type 1 and type 2 DM across a wide age range, without stratified analyses; future research should stratify results by DM type and age to explore potential differences in outcomes. Variability between devices limits the establishment of universal reference values, and consistent use of the same device is recommended for longitudinal patient monitoring.

## 5. Conclusions

In this systematic review, we examined the diagnostic accuracy and reliability of VPT measurement devices used for the detection of DPN and synthesized evidence from 18 studies. Based on the integrated findings, we conclude that VPT measurement devices are a useful screening tool for the detection of DPN; however, their diagnostic accuracy and reliability are not entirely consistent and largely depend on technical and methodological factors. To improve clinical applicability, standardization of diagnostic threshold values and measurement procedures is needed. Future studies directly comparing multipoint and single-point testing protocols would be valuable; if comparable diagnostic accuracy is demonstrated, a single-point approach would be preferable, as it would allow substantial time savings for healthcare professionals.

## Figures and Tables

**Table 1:** Risk of bias assessment of diagnostic accuracy studies using the JBI Critical Appraisal Checklist.

ITEM	[5]	[6]	[15]	[27]	[28]	[30]	[26]	[29]
1. Was a consecutive or random sample of patients enrolled?	U	U	U	U	Y	U	Y	U
2. Was a case–control design avoided?	Y	N	Y	N	N	Y	Y	N
3. Did the study avoid inappropriate exclusions?	Y	Y	Y	Y	Y	Y	Y	Y
4. Were the index test results interpreted without knowledge of the results of the reference standard?	U	U	U	U	Y	U	U	U
5. If a threshold was used, was it pre-specified?	Y	Y	Y	N	Y	Y	Y	Y
6. Is the reference standard likely to correctly classify the target condition?	Y	Y	Y	U	Y	Y	Y	Y
7. Were the reference standard results interpreted without knowledge of the results of the index test?	U	U	U	U	U	U	U	U
8. Was there an appropriate interval between index test and reference standard?	Y	U	Y	Y	Y	Y	Y	U
9. Did all patients receive the same reference standard?	Y	Y	Y	Y	Y	Y	Y	Y
10. Were all patients included in the analysis?	Y	Y	Y	Y	U	Y	Y	Y

Y, yes; N, no; U, unclear.

**Table 2:** Risk of bias assessment of studies assessing reliability using the QAREL checklist.

ITEM	[37]	[33]	[34]	[38]	[39]	[35]	[31]	[40]	[32]	[36]
1. Was the test evaluated in a sample of subjects who were representative of those who the authors intended the results to be applied?	Y	Y	Y	Y	Y	Y	Y	Y	Y	Y
2. Was the test performed by raters who were representative of those to whom the authors intended the results to be applied?	Y	U	U	N/A	N/A	Y	N/A	N/A	U	N/A
3. Were raters blinded to the findings of other raters during the study?	Y	N/A	N/A	N/A	N/A	Y	N/A	N/A	Y	N/A
4. Were raters blinded to their own prior findings of the test under evaluation?	Y	N/A	U	N/A	N/A	Y	N	N/A	N/A	N/A
5. Were raters blinded to the results of the reference standard for the target disorder (or variable) being evaluated?	N/A	N/A	U	N/A	N/A	N/A	U	U	N/A	U
6. Were raters blinded to clinical information that was not intended to be provided as part of the testing procedure or study design?	U	Y	U	N/A	N/A	Y	Y	Y	N	N/A
7. Were raters blinded to additional cues that were not part of the test?	U	N	N	N/A	N/A	N	N/A	N/A	N/A	N
8. Was the order of examination varied?	N/A	U	Y	N/A	N/A	Y	N/A	N/A	N/A	N/A
9. Was the time interval between repeated measurements compatible with the stability (or theoretical stability) of the variable being measured?	Y	Y	Y	Y	Y	Y	Y	N	Y	Y
10. Was the test applied correctly and interpreted appropriately?	Y	Y	Y	Y	Y	Y	Y	Y	Y	Y
11. Were appropriate statistical measures of agreement used?	Y	Y	Y	Y	Y	Y	Y	Y	Y	Y

Y, yes; N, no; U, unclear; N/A, not applicable.

**Table 3:** Characteristics of participants included in the studies.

Study	Sample Size	Sex (M/F)	Age	Type of DM	Duration of DM (Years)	DPN (%)
[37]	100	58/42	57 ± 8	Type 1: 8, type 2: 92	12 ± 7	100
[33]	42	NR	NR	NR	NR	100
[34]	90	56/34	65.64 ± 8.65	Type 2	9.96 ± 8.83	NR
[38]	26	NR	NR	NR	NR	58
[39]	20	G1 (DPN & ulcers): 5/5; G2 (Charcot foot): 4/8; G3 (autonomic neuropathy): 5/5; G4 (painful neuropathy): 6/9	G1: 42.5 (35–52); G2: 36.9 (26–55); G3: 31.7 (24–43); G4: 35.9 (20–52)	G1: type 1: 7, type 2: 3; G2: type 1: 12; G3: type 1: 10; G4: type 1: 14, type 2: 1	G1: 16.4 (7–30) G2: 21.7 (12–37) G3: 15.5 (8–26) G4: 14.7 (3–42)	100
[27]	242	162/80	48.80 ± 11.29	Type 2	5.69 ± 5.57	55
[35]	50 (inter), 44 (intra)	33/17	M: 72 ± 11, F: 73 ± 7,	Type 2	NR	32
[15]	421	DPN: 149/78 non-DPN: 103/91	DPN: 62.1 ± 13.4 non-DPN: 54.3 ± 15.8	Type 2	DPN: 10.0 (5.3–17.0) non-DPN: 6.0 (1.0–11.0)	54
[31]	118	58/60	13.5 ± 3.4 (range: 8–20)	Type 1	5.7 ± 3.5 (range: 2–16)	3
[40]	16	NR	NR	Type 1 and 2	NR	NR
[5]	562	274/288	56.42 ± 12.57	Type 2	5.93 ± 4.75	64.6
[6]	30	16/14	53.43 ± 5.59	Type 2	NR	100
[32]	15	9/6	62.1 ± 8.4	NR	NR	100
[36]	85	60/15	61 (21–82)	Type 1: 26, type 2: 59	Mean: 12 (range 1–26)	NR
[28]	307	144/163	13.3 ± 4.6	Type 1	5.1 ± 4.9	6
[30]	48	NR	51.31 ± 7.89	Type 2	5.95 ± 4.81	29
[26]	100	48/52	52.9 (32–80)	Type 2	6.9 (0–30)	71
[29]	30	16/14	53.43 ± 5.59	Type 2	NR	NR

NR = not reported; G = group; DM = diabetes mellitus; DPN = diabetic peripheral neuropathy.

**Table 4:** Diagnostic accuracy of VPT measurement devices.

Study	Instrument	Measurement Site	Cut-Off Value	Reference Standard	Results
[5]	Vibrasense (Ayati DevicesPrivate Ltd., Mumbai, India)	Six plantar sites (hallux; heads of 1st, 3rd, 5th metatarsals; medial midfoot; heel); mean VPT	VPT ≥ 15 V	NCS	Sens.: 82.14% Spec.: 78.79% AUC: 0.839 (95% CI: 0.730–0.948, *p* < 0.001)
[15]	Neurothesiometer (Beijing Laxons Technology Co., Ltd., Beijing, China)	Distal pulp of left hallux; mean of three trials	VPT > 14.9 V	1. Physician-diagnosed DPN 2. Nerve conduction studies (NCS) 3. Confirmed DPN (presence of neuropathic symptoms together with abnormal NCS)	1. Spec.: 85.1%, Sens.: 67.0%, AUC: 0.804 2. Spec.: 77.0%, Sens.: 66.5%, AUC: 0.761 3. Spec.: 76.6%, Sens.: 67.2%, AUC: 0.766
[27]	Biothesiometer (China Beijing Di Meide Technology Co., Ltd., Beijing, China)	Hallux pad. Three measurements. The test was repeated three times on each foot, and the average VPT value was recorded for both feet.	VPT > 14.05 V	Established diagnosis of DPN; 1. MNSI symptom score ≥ 4, or 2. MNSI examination (signs) score ≥ 2 and NDS ≥ 6, or 3. NDS ≥ 3 and MNSI symptom score ≥ 4.	Sens.: 80% Spec.: 92% AUC: 0.941
[6]	Biothesiometer	Six points on the plantar surface of both feet.	VPT > 25 V	NCS	Sens.: 70%, Spec.: 86.67%
[28]	Biothesiometer (Bio-medical Instrument, Newbury, OH, USA)	The medial malleolus and the plantar surface of both great toes. Three measurements were taken at each site.	97th percentile of healthy controls	NCS	Sens.: 80% Spec.: 76%
[30]	Biothesiometer (Diabetic Foot Care India Private Limited, Chennai, India)	at the distal interphalangeal (DIP) joint of both great toes	VPT > 15 V; 25 V	NCS	VPT > 15 V: Sens.: 78.6% Spec.: 52.9% VPT > 25 V: Sens.: 50% Spec.: 91.2%
[26]	Biothesiometer (Sensitometer, Dhansai Lab, Mumbai, India)	At six sites on the plantar surface of both feet—the great toe, first metatarsal head, third metatarsal head, fifth metatarsal head, midfoot (arch), and heel.	VPT > 15 V	NCS	Sens.: 86% Spec.: 76%
[29]	Sensiometer, Dhansai laboratory, Mumbai, India	The great toe, first metatarsal head, third metatarsal head, fifth metatarsal head, midfoot arch, heel, and posterior tibial arch of both feet	VPT > 25 V	NCS	Sens.: 70% Spec.: 86.67%

VPT—Vibration Perception Threshold, NCS—Nerve Conduction Study, MNSI—Michigan Neuropathy Screening Instrument, NDS—Neuropathy disability score.

**Table 5:** Inter-rater reliability of VPT measurement devices.

Study	Instrument	Measurement Site	Inter-Rater Reliability
[37]	Biothesiometer	Hallux base	Right: κ = 0.65 (95% CI 0.46–0.84); Left: κ = 0.58 (95% CI 0.40–0.70)
[35]	Neurothesiometer (Wilford Industrial, Nottingham, UK)	Right great toe tip; three measurements; mean of three values.	κ = 0.61 (95% CI 0.45–0.77), *p* < 0.01
[40]	Neurothesiometer	Three measurements were taken at the tip of the hallux on both feet, and the highest average VPT value from either foot was used for analysis	κ = 0.51
[32]	Maxivibrometer (LDS 203; Ling Dynamic Systems Ltd., Royston, UK), Biothesiometer (Bio-Medical Instrument Co.)	The great toe and heel of the right foot; three measurements were taken.	ICC: 0.96 (Maxivibrometer); ICC: 0.93 (Biothesiometer)

**Table 6:** Intra-rater reliability of VPT measurement devices.

Study	Instrument	Measurement Site	Intra-Rater Reliability
[37]	Biothesiometer	Hallux base	Right: rater A: κ = 0.51, rater B: κ = 0.57 (95% CI 0.40–0.68); left: rater A: κ = 0.64, rater B: κ = 0.51 (95% CI 0.44–0.71)
[33]	Vibratron II (Physitemp Instruments, Clifton, NJ, USA), Neurothesiometer (Scientific Laboratory Supplies, Nottingham, UK)	Hallux bilaterally; three trials	Vibratron II CV (%) = 31–34, Neurothesiometer CV (%) = 6–8
[34]	Vibratron II (Physitemp Instruments, Inc.: Clifton, NJ, USA)	Hallux bilaterally	Vibratron II ICC = 0.958 (95% CI 0.94–0.98)
[38]	Biothesiometer	Dorsal hallux near nail bed; lateral malleolus	hallux: CV (%) = 16.5 ± 5.8 (4–21); malleolus: CV (%) = 18.6 ± 9.5 (4.4–28)
[39]	Biothesiometer (Biomedical Instruments, Newbury, OH, USA)	The palmar surface of the distal phalanx of the dominant index finger and the tip of the hallux; the result was calculated as the mean of three measurements.	r = 0.91, *p* < 0.01
[35]	Neurothesiometer (Wilford Industrial, Nottingham, UK)	Right hallux tip; three measurements; mean of three measurements	κ = 0.52 (95% CI 0.21–0.82) *p* < 0.01 to κ = 0.78 (95% CI 0.58–0.98) *p* < 0.02
[31]	Biothesiometer	The pads of the thumb and index finger, the malleolus, and the pad of the hallux. Two measurements were taken at each site. All measurements were performed bilaterally	hallux (left): κ = 0.69 (±0.05), hallux (right): κ = 0.64 (±0.05), tibia (left): κ = 0.70, tibia (right): κ = 0.64
[36]	Biothesiometer (Arnold Horwell, London, UK) Neurothesiometer	At the tip of the hallux. For each foot, the mean value of three measurements was calculated	Biothesiometer: CV (%): 8.6; Neurothesiometer: CV (%): 8.1

## Data Availability

No new data were created or analyzed in this study. Data sharing is not applicable.

## Supplementary Materials

The following supporting information can be downloaded at: https://www.mdpi.com/article/10.3390/diagnostics16020217/s1, PRISMA checklist. Reference [65] is cited in the supplementary materials.

## Abbreviations

The following abbreviations are used in this manuscript:

NCS	Nerve conduction studies
DPN	Diabetic peripheral neuropathy
DM	Diabetes mellitus
VPT	Vibration perception threshold
